# Oleogels in Food: A Review of Current and Potential Applications

**DOI:** 10.3390/foods9010070

**Published:** 2020-01-08

**Authors:** Andreea Pușcaș, Vlad Mureșan, Carmen Socaciu, Sevastița Muste

**Affiliations:** 1 Department of Food Engineering, Faculty of Food Science and Technology, University of Agricultural Sciences and Veterinary Medicine Cluj-Napoca, 400372 Cluj-Napoca, Romania; andreea.puscas@usamvcluj.ro (A.P.); sevastita.muste@usamvcluj.ro (S.M.); 2 Department of Food Science, Faculty of Food Science and Technology, University of Agricultural Sciences and Veterinary Medicine Cluj-Napoca, 400372 Cluj-Napoca, Romania; carmen.socaciu@usamvcluj.ro

**Keywords:** oleogel, structured lipids, edible organogel, trans and saturated fats alternatives

## Abstract

Legislative limitations of the use of trans and saturated fatty acids, the rising concerns among consumers about the negative effects of some fats on human health, and environmental and health considerations regarding the increased use of palm fat in food and biodiesel production drove to innovations in reformulating fat-containing food products. Oleogelation is one of the most in-trend methods for reducing or replacing the unhealthy and controversial fats in food products. Different edible oleogels are being formulated by various techniques and used in spreads, bakeries, confectioneries, and dairy and meat products. This review exclusively focuses on up-to-date applications of oleogels in food and mechanisms of gelation, and discusses the properties of new products. Research has produced acceptable reformulated food products with similar technological and rheological properties as the reference products or even products with improved techno-functionality; however, there is still a high need to improve oleogelation methods, as well as the technological process of oleogel-based foods products. Despite other strategies that aim to reduce or replace the occurrence of trans and saturated fats in food, oleogelation presents a great potential for industrial application in the future due to nutritional and environmental considerations.

## 1. Introduction

Fats and oils are mainly triglycerides containing monounsaturated, polyunsaturated, and saturated fatty acids, besides several minor compounds. In most of the cases, food products contain a mixture of these triglycerides. It was considered that fats may have a bad influence on consumer health and low-fat diets were highly promoted, but recent studies have shown that only trans and saturated fats are associated with cardiovascular disease occurrence [1,2]. In foods, saturated and trans fatty acids play a technological role, since they are responsible for some specific properties such as flavor, palatability, and texture [3]. Moreover, the triacylglycerols assemble into a supracolloidal network, transforming fats into solid or solid-like materials, thereby conferring structure to food products [4].

Functional lipids and nutritional improvement of lipid-containing foods have gained more and more interest since (i) recent regulations banned the use of artificial trans fats and (ii) several entities suggested the limitation of saturated fats in foods. The World Health Organization (WHO) recommends the amount of total, saturated, and trans fats, to be less than 30%, 10%, and 1% of the total energy intake, respectively [5]. Concerning the issue of trans fats, the Food and Drug Administration (FDA) released in 2015, its final determination [6] that partially hydrogenated oils, the main source of artificial trans fats in processed foods, are not generally recognized as safe (GRAS). The manufacture of foods containing specific (limited petitioned) partially hydrogenated oils was stopped on 18 June 2018, in order to clear distribution by 1 January 2020. WHO started a campaign named ”REPLACE” that aims to review the dietary sources of industrially produced trans fats, encourage and promote their replacement with healthier fats and oils, adopt regulatory laws and actions to eliminate industrially produced trans fats, assess and check the trans-fat content in the food supply, observe consumers’ preference, create awareness of the implication of fats in health, and enforce compliance with policies and regulations [7]. The European Food Safety Authority (EFSA) approved a report on 8 June 2018 in order to depict the current goals and recommendations for consumers related to the intake of trans fats and health issues, concluding that there is an effect dependent on the dose that increases the risk of cardiovascular heart disease as compared to the intake of other fatty acids in the diet [8]. Moreover, the European Commission (EU) imposed a limitation on the use of trans fats to 2 grams per 100 grams of total fat (trans fats naturally occurring in the fat of animal origin are not included in this limitation), as stated on the very recent Regulation No. 649 from 24 April 2019 [9].

The current practice used in the food industry for trans-fat issues is the replacement of trans fats with natural saturated fats, generally obtained by the fractionation of different tropical oils, mainly palm oil. The use of palm oil as the main source of saturated fats might affect both the consumers’ health and environment. Palm oil is widely used in the food industry due to considerations such as low purchasing prices, high stability against oxidation processes, and long validity term. The high prevalence of palm fat in food products leads to high consumption levels of saturated fats, a fact that arises some health concerns—the consumption of saturated fats (except for stearic acid) influences cholesterol levels in blood and leads to cardiovascular diseases [2]. Some deforestations due to increased palm oil usage in both food industry and biodiesel manufacturing were reported [10]. Numerous palm tree plantations which lack certification were identified in certain areas, where tropical deforestation and the release of CO_2_ due to palm oil production are high [11]. Therefore, there is a real need for healthier, trans fatty acid-free, stable, and solid-like fats, which maintain their structure at ambient temperature, assuring a longer shelf-life [12].

Consequently, food specialists from research and industry must find solutions for the replacement of trans and saturated fatty acids from foods without altering their processing characteristics and technological or sensorial qualities, while maintaining the interest of the consumers, their demand for the products, and complying with the abovementioned regulations.

Although there are some ingredients that are used as fat replacers, such as (i) fat substitutes (e.g., different sucrose fatty acid polyesters), (ii) mimetic fats, which can be carbohydrate-based (e.g., gums, dextrin, maltodextrins, polydextrose, and derivatives of cellulose, starch, and oat flour) or mimetic fats of protein origin (e.g., egg white, lactalbumin, lactoglobulins, whey, soy, or wheat proteins) [13], (iii) fat extenders, and (iv) low calorie fats [14], there are some major insufficiencies for their application. For instance, fat substitutes are specific synthetic molecules that provide no energy calories, but are not capable of reproducing the taste properties of fat. Fat mimetics, generally polar, water-soluble compounds, are prone to denaturation in high-temperature applications, such as frying; moreover, due to their polar characteristics, their lipid-soluble flavor-carrying capacity is altered, as compared to conventional fats [13].

Vegetable liquid oils can be tailored to be solid-like fats; partial hydrogenation, fractionation, or interesterification were exploited until health concerns related to artificial trans fats, as well as highly saturated fats, occurred. From the abovementioned techniques that can impart solid-like properties to liquid oils, enzymatic interesterification is a promising method, which, regardless of the high cost of enzymes, could produce final products with zero trans fatty acids and without affecting the sensorial properties [15]. While partial hydrogenation is considered to be the main process that leads to the formation of artificial trans fatty acids, other methods bring a high content of saturated fats in the final food product composition, increasing the risk of developing cardiovascular diseases, leading to obesity and diabetes [3].

Food manufacturers must take into consideration the use of oleogelation, a recent technique of converting liquid vegetable oil into a solid-like gel with the use of organogelators, explored by researchers in order to create novel food ingredients, which may have the functionality of fats and the nutritional profile of liquid oils [16,17,18,19,20]. Oleogels can be formed from a wide range of structuring agents that will lead to different gelation mechanisms, occurring on a nano- and micro-scale and that induce specific macroscopic features (e.g., rheological/textural). In addition, the term “oleocolloids” refers to a complex system, like a dispersion of a medium (water/air) in the oil, resulting in emulsions or foaming oleogels, or even systems like oil-in-water-in-oil (O/W/O) emulsions or layered structural matrices (oleofilms) [21,22]. Based on their molecular weight, oleogelators are classified in low-molecular-weight compounds, or polymeric gelators [4]. The structuring agents are either non triacylglycerolic oleogelators, more specifically crystalline particles or self-assembling structures, self-assembled fibrillar networks, emulsions, polymers, inorganic compounds, or lipid-based gelators such as waxes, fatty acids, fatty alcohols, or monoglycerides [4,19]. Different oleogel formulations and their characterization have already been intensively reviewed and the available oleogels and their use as conventional fat replacers have been systematically presented (Table 1). More explorative research is focusing on studying the synergism of some organogelators or some blends in creating ideal oleogels that not only have good stability, but can bring additional benefits to human health or to the environment. A mixture of oleogelators could allow a specific tailoring of oleogels behavior and increase the possibility of their usage in food products. By varying the ratios of different components of a synergistic mixture, numerous systems can be developed; therefore, it is possible to tune the gel characteristics by changing different variables or processing conditions of the system. A mixture of oleogelators may also co-assemble and form nanoscale gel phases (randomly, specifically, or alternatively) [23]. Some mixtures of the oleogelators displayed synergistic effects when they were investigated, such as sterols (*γ*-oryzanol and *β*-sitosterol), fatty acids and fatty alcohols, monoglycerides, and phytosterols. High sensitivity to water remains a technical challenge of exploiting phytosterol oleogels and in order to facilitate their incorporation into the food matrix, two different approaches were proposed—addition of lecithin or glycerol [24]. Synergistic interactions between and organogelator and an additional component have already been randomly discovered. Stearic acid combined with Span 60 has shown a modified habit of crystallization. Co-crystallization and the creation of a mixed structure were observed in the addition of tripalmitin to candelilla wax, and a sequential crystallization resulted when high and low melting waxes were used together as a mixture of organogelators. Another component that can act as a crystal habit modifier is lecithin, with several synergic interactions already reported [25,26].

Innovative food ingredients were developed based on low energy by-products with health benefits. More specifically, 25% micronized agri-food residues of tomato (the peels) or spent coffee grounds, in suspension, were dispersed in peanut oil, resulting in the formation of a capillary suspension (spanning network) when a secondary immiscible fluid was added (water). The results are suggesting different approaches in structuring vegetable oils and transforming them to oleogels [27]. Even the combination of phenolic ester particles and polymers was explored so as to create the oleogels. Oleogels of camellia-oil were developed with tea polyphenol-palmitate particles and varying citrus pectin concentration. The oleogelation was possible with the emulsion-template method. Firstly, an oil in water emulsion was prepared, stabilized by tea polyphenol-palmitate particles and citrus pectin as a template, followed by the removal of the water phase to form a dried product through freeze-drying, with the shearing of the dried product and resulting in the camellia oil-based oleogel [28].

Novel oleogels were produced based on fermentation implying residues from the food industry side streams, namely sugarcane molasses and soybean processing side streams, which were fermented by the oleaginous yeast *Rhodosporidium toruloides* to produce microbial oil. Wax esters, which were used as organogelators, were the result of enzymatic catalysis of the microbial oil or soybean fatty acid distillate. Fermentation was conducted either without any addition of trace elements and phosphate salts, or with simultaneous addition of nitrogen sources, trace elements, and phosphate salts, in this case generating the highest lipid concentration. Soybean cake hydrolysate constituted the perfect medium for yeast growth, so the microbial oil production was efficiently carried out. The growth of *R. toruloides* is possible in mediums containing phosphorus. In shake flask, the fermentation resulted in microbial oils rich in oleic acid (50.6–56.6%), palmitic acid, stearic acid and linoleic acid. The highest oleic acid amount resulted when the fermentation was conducted in a medium that contained trace elements and phosphate salts supplements, while the highest linoleic acid content (6.7%) was observed when the medium was deprived of nitrogen [29].

**Figure 1:**
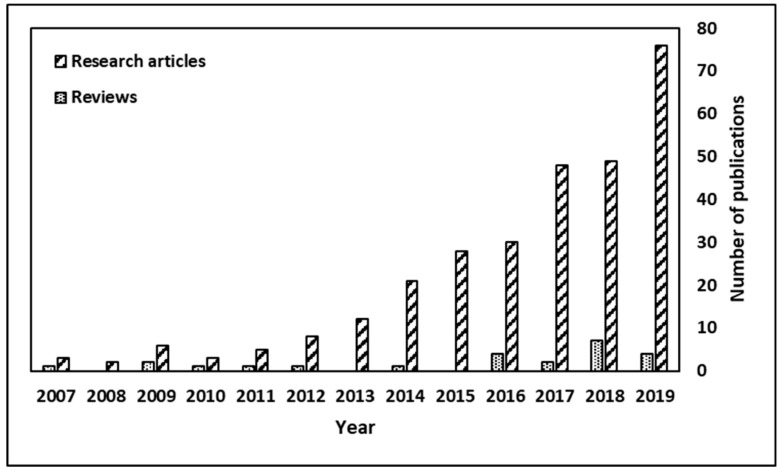
The increasing number of articles for oleogelation topic; searching the ISI Web of Science Core Collection Clarivate Analytics by the keywords « “food” and “organogels” or “oleogels” » (updated to 13 November 2019).

The oleogelation subject is of high interest worldwide: searching the ISI Web of Science Core Collection Clarivate Analytics (13.11.2019) by the keywords combination « ”food” and “organogels” or “oleogels” » on Topic fields (i.e., title, abstract, author keywords, and keywords plus) revealed 353 results; when the search was refined by selecting the food relevant ISI WoS categories (i.e., food science technology, chemistry applied, chemistry multidisciplinary, nutrition dietetics, biochemistry molecular biology, and chemistry physical), 300 results were shown, out of which 24 are review-type articles. Figure 1 depicts the increasing number of articles (research and review types) for oleogelation topic during the last years, while Table 1 introduces the books and the main reviews published on the same theme. The complex approach in the study of oleogelation led to the publication of four books [30,31,32,33], which extensively discuss topics like the health-related issues of trans and saturated fats, oil structuring and oleogelation techniques that may help the decrease of unhealthy fats in foods.

Due to the existence of various organogelators and oleogelation techniques, there are numerous publications describing different formulations of oleogels and their rheological behavior, the solid fat content, the thermal behavior, their microscopic structure and textural properties. These characteristics are of utmost importance for assessing the compatibility of organogelators with the vegetable oils involved in oleogelation, in selecting the most suitable oleogels for specific processing conditions of food and in order to understand some of the possible influences over oleogel formation that may occur due to food composition and other food ingredients. The type of vegetable oil involved in the formation of the oleogel must also be well-chosen, since recent research demonstrate that its composition, the carbon chain length of the fatty acids and the level of unsaturation of the fatty acids, are factors influencing the oleogel properties and its behavior in food matrices [22].

In order to be used in food products formulation, organogelators must be readily available, effective in transforming liquid oils into solid-like systems, even if they are used in very low percentage, they must be cost effective and easy to store, they should not require any additional processing and last, but not least, organogelators must present approval for use in edible products or ideally a GRAS status [19,20]. Besides this, it is important to note the capability of oleogelators to mimic the physical properties of the replaced fats and to display desired functional properties in food products (structure, texture, fracturability, and glossy appearance). Fat is the pillar of some food products’ internal network, and when it is replaced, it is highly desirable for the novel compounds to impart the fats’ specific properties to the food product. Textural characteristics are important quality properties of the high-fat food; texture parameters become critical when considering trans and saturated fats substitution by oleogels. Changing the consumers’ habits for opting for nutritionally valuable food products should be done without sacrificing the pleasurable taste, and mouthfeel imparted by solid fats.

In conclusion, interesting practical applications might be developed, since it is possible to formulate oleogels with various mechanical properties and structural strengths by identifying the optimum lipid structurants classes and compounds, their ratio, the assembling strategies, processing parameters of structuring agents in accordance to the type of liquid oil used and the food product aimed to be improved. In the near future, in order to comply with the new regulations, fat from several food products might be substituted by tailor-made oleogels.

## 2. Oleogels—Current Applicability in Food

Oleogels have been applied in the development of many food products, such as spreads, bakery products, sweets, dairy, and meat products, not only for the replacement of trans and saturated fats but also because oleogels demonstrated other important roles as carriers for water-insoluble bioactive substances, stabilizers in emulsifier-free products, oil binding, or imparting heat resistance to the product. Current food applications of oleogels are summarized in Table 2.

### 2.1. Breakfast Spreads

Margarines represent a spread alternative and butter substitute but, generally, its composition included hydrogenated fats, translating into high amounts of saturated fatty acids. Margarine containing water, milk, additives, and lipids are hardened oils consumed directly as breakfast spreads or products used as ingredients in different processed foods, such as bakery products. Consumers are recently avoiding the consumption of margarine or margarine-including products because of saturated fatty acids content. The processing conditions and the type of fatty acids involved in the formulation of the breakfast spreadings are responsible for the spreadability, texture, consistency, and flavor. The melting points of the fat are responsible for the functionality of spreads: spreadability and firmness (2–25 °C), mouthfeel and waxiness (30–40 °C). The shape and stability of fat crystals developed during crystallization account for the texture and appearance of spreads; if large crystals result during storage, a sandy mouthfeel may occur, thus small crystal formation is desired. Flavoring compounds are usually lipophilic, and they are released by fats with a good melt-off. Numerous studies propose the usage of oleogel emulsions as breakfast spreads and aim to promote new consumption habits for healthier oil containing products. The feasibility of 12 different vegetable oils was explored in order to develop a healthy margarine formulation using 3%, 5%, and 7% sunflower wax as potential structuring agent, relative firm margarines being obtained. The water in oil emulsions prepared from 3% sunflower wax oleogels resulted in greater firmness than the references, whereas margarines formulated with 7% sunflower wax oleogels displayed a decreased firmness. However, the melting behavior of oleogel containing water in oil emulsions was more prominent than the commercial spread and margarine products. The firmness of the new formulation of oleogel margarine decreased with the increase of the amounts of polar compounds content of the vegetable oils used for oleogel formation. Interestingly enough, the fatty acid compositions of the oil did not influence firmness. It was concluded that more studies are needed for revealing the factors affecting the physical characteristics of the oleogels and the new margarine formulation, since other factors, such as other minor components in oil or different obtaining methods of the oleogelators, the position of fatty acids in the glycerol backbone, and the occurring interaction between the ingredients, seem to affect the firmness of margarine [49].

Plant waxes, including sunflower wax, rice bran wax, and candelilla wax, were used to structure soybean oil and test the suitability for their incorporation into margarine. The properties of the newly formulated margarines could also be influenced by the structure of the organogelator. The firmness of the margarine containing soybean oil gelled with sunflower wax in different percentages (2–6%) was similar to that of the reference margarine which contained 18–30% hydrogenated soybean oil and high in saturated fats. Candelilla wax oleogel showed phase separation and rice bran wax margarine presented low firmness, so they were not suitable for developing new breakfast spreads [50]. Oleogels based on sunflower oil and 2% shellac were prepared and have been used in the formation of water-in-oil emulsions. These emulsions were prepared with up to 60% water, without any other emulsifiers. The dispersion of water in the melted oleogels was possible due to the long-chain fatty alcohols from shellac, which acted as surface active components. When the emulsions were cooled down, further stabilization occurred due to the crystallization of shellac wax in the entire mass of the emulsion, as well as in the interface. The shellac oleogel spread was stable for over 4 months of storage [51]. Even if currently shellac has a GRAS status, and is used as coating/glazing agent, further approvals for its use in higher amounts and different food applications are required. Virgin olive oil oleogels with 3%, 7%, or 10% carnauba wax or with monoglycerides as oleogelators were also analyzed to determine the most suitable oleogel as a spreadable product [52]. After thermal, textural and morphological characterization, it was concluded that the 7% monoglycerides containing virgin oil oleogel have textural and thermal properties similar to commercial breakfast margarines. The lipid oxidation of the stored samples at room temperature and refrigeration conditions was measured monthly, over a period of 3 months, by the peroxide value; it was only the storage time which determined a gradual increase of oil oxidation [52].

Oleogel emulsions were prepared through simultaneous oleogelation and emulsification from virgin olive oil and 3.75% or 4.5% beeswax, with the addition of Tween 20 or Tween 80 as emulsifiers. The developed oleogels textural values, such as firmness and adhesiveness, were moderately comparable to breakfast margarine. No changes of texture, color, and oxidation resulted during the 3-month storage. The emulsions formed with oleogels, with slight improvements and further optimization, represent a potential formulation for margarine or shortening replacement in bakery products [53].

### 2.2. Confectionery

The fat phase has the greatest impact on the quality of chocolate, compound, fillings, and related confectionery products. The two main fats groups used in chocolate manufacturing are cocoa butter or its replacers and milk fat; these fats are responsible for the hardness, ability to temper, and melting point of the chocolate products [54]. In average, a chocolate product can contain up to 20% saturated fats so healthier formulations are highly desired [55].

#### 2.2.1. Chocolate and Chocolate Pastes

Chocolate and chocolate spreads are composed by a predominant fat medium with dispersed cocoa and sugar particles. Chocolate pastes display characteristics of both liquid-like materials and solids at the same time because of their rheology [56]. Usually, solid fats (cocoa butter and palm oil) or hydrogenated vegetable oils are used for the preparation of the chocolate paste formulations, despite the high levels of saturated fatty acids. In order to avoid oil exudation during storage, an oil binder, usually based on hydrogenated fats (high melting triglycerides), is incorporated [57]. Shellac oleogel was used to completely replace the oil binder on a 1:1 ratio in a chocolate paste. Moreover, palm oil was also partially replaced by 27% liquid rapeseed oil which, in the presence of shellac wax, formed an oleogel. The samples did not show any oil separation during four weeks of storage; therefore, shellac wax could act as an oil binder [51]. The fat phase in chocolate cream was replaced with a 1:1 mixture of palm oil and pomegranate seed oil oleogel structured with 5% of different types of organogelators: monoglycerides, beeswax or propolis wax [58]. The use of waxes led to a weak structure formation and to polymorphism or post crystallization of oleogels, so it affected the firmness of chocolate during storage. Oleogel and palm oil mixtures displayed good oil binding properties, thus being a feasible option for fat phase substitution in chocolate spread formulation when attention is given to the chemical compatibility between the chosen organogelators and palm oil [58]. Hybrid hydrogels were developed as a novel approach to produce heat resistant, low-fat chocolate. Sodium alginate and pectin were used to prepare a hybrid hydrogel, the citric acid being the cross-linker; the developed network was the result of physical and chemical cross-linking. Dispersions of hydrogel in chocolate (up to 50%), showed less surface roughness, while exhibiting a glossy appearance and high melting resistance at 80 °C. However, due to the increased water content in the formulation, the stability during storage might be affected [59].

#### 2.2.2. Pralines

Oil migration phenomenon might occur in pralines and is considered the main cause of chocolate bloom. Soybean oleogels were investigated as possible agents of hindering oil migration in commercial confectionery filling for praline products. Soybean oil was gelled with monoglycerides (1%, 3%, or 6%) or an equimolar mixture of sorbitan tri-stearate and lecithin (6%, 8%, or 10%). Oleogels displayed time-dependent shear thinning properties, indicating that it is possible to hinder migration of fat fillings and showing good flowing capacity through the equipment. Storage did not affect the texture of oleogels, good structural stability being noticed [63].

The 10% or 25% sterol structured organogels based on *γ*-oryzanol and *β*-sitosterol 1:1 mixture were included in pralines to inhibit or prevent the migration of oil phase. The inclusion level of the oleogel in the praline system was either 2.5% or 14%. The oil migration was assessed during 6 months of storage at different temperatures (10, 18, and 28 °C) via thermal analysis of a surface sample. The proposed system consisting of three layers (i.e., chocolate, gel, and nougat) and the gelled sample of the chocolate are reported as promising solutions to either inhibit the migration of oil phase or improve the nutritional fingerprint by liquid oils incorporation. Namely, limited oil migration was demonstrated for the samples stored at 18 °C. Remarkably, during storage at 18 °C, the sample with a gelled nougat layer with 2.5% organogel showed no oil migration. The strongest oleogels (25% structurants) showed the best oil migration inhibition when samples were stored at the most challenging temperature (28 °C). Even more promising results were obtained with gelled chocolate system; when including a ratio of 2.5% of oleogel (25% structurants) in the chocolate phase, the relative migrated oil level was reduced by 50% [62].

#### 2.2.3. Fillings

Palm oil replacement was assessed with the usage of beeswax-oleogel for decreasing the amount of saturated fat in hazelnut filling. A novel low-saturated fat confectionery filling was designed, based on the replacement of palm oil with beeswax oleogel in the hazelnut filling, at replacement levels of 17%, 33%, and 50%. Three types of oleogel containing hazelnut fillings resulted. Besides the fat phase, the fillings contained sugar, and dispersed hazelnut particles [64]. The crystallization and gelling behavior of the blends containing palm oil and wax-based oleogels and the crystallization of the novel types of filling were analyzed. The filling containing 17% of palm oil replaced with wax oleogel displayed the same strength as the reference containing 100% palm oil. However, further research needs to be conducted in order to analyze the influence of different chemical components of waxes, on the gelation mechanism and crystallization behavior of wax-based palm-blends designed to lower the saturated fats in confectionery products.

### 2.3. Pastry

Baked food products, such as crackers, cakes, muffins, and biscuits, typically contain high amounts of solid fats, the saturated fat content reaching levels of 31.8% for baking and frying fats, 7.4% in cakes, 21% in cookie creams, and 4.1% in crackers [55]. In bakery, the role of fat may differ, but fat is mainly involved in the formation of a microstructure and is responsible for the stabilization of air cells in the dough. For high fat products, the consistency of fat at dough making temperature, together with sensory properties, such as flavor release, are important for the quality of the final product. During storage, fat may also influence oil migration and oxidative stability. Normally, yeast-raised products (bread group) are characterized by a low-fat content; however, fat is important for the lubrication of gluten, being indispensable for the desirable, soft texture of bread. In laminated products, fat with suitable plasticity and high firmness is needed for spreading between the layers of the dough during the several sheeting and folding operations. Cakes are formulated with fats because, used together with emulsifiers, they produce the desired aeration properties of the batters, resulting in a softer and crumb texture. Doughs for biscuits and cookies demand plastic fats for forming. An important property of the fat is the crystallization behavior after baking, because fat-bloom on the surface of the biscuits is not desired.

#### 2.3.1. Cookies and Biscuits

Biscuits and cookies are two of the most popular bakery items consumed all over the world, whose main ingredients are viscous enough to allow the pieces of dough to be baked on a flat surface. In biscuits composition, sugars and solid fats are usually included in large amounts. The reduction of saturated fat with oleogel and shortening blends in baked cookie formulations was proposed with the usage of 3% or 6% candelilla wax containing oleogels [65]. Generally, important amounts of saturated fatty acid can be reduced in baked products by replacing shortening with oleogels, but the real challenge remains the maintenance of desired physical properties. When the oleogels or the oleogel/shortening blends were subjected to shear, their structure was transformed from solid-like to viscous liquid. A higher amount of shortening in the fat blends resulted in better shear sensitivities of the samples. The analysis conducted on the dough containing oleogel revealed a better consistency and low levels of hardness in comparison with the reference [99]. The textural properties of a mixture of shortening and oleogel were more similar to the reference. The physical properties of cookies were improved when the oleogel was added to the composition, compared to the replacement of fat with the liquid oil, but the oleogel did not achieve the functionality necessary for replacing fat completely; thus, the formation of some blends is highly recommended.

New cookies’ formulation from vegetable oil organogels were formulated with four different waxes, including sunflower wax, rice bran wax, beeswax, and candelilla wax and three types of oil: olive oil, soybean oil, and flaxseed oil, that are rich in oleic acid, linoleic acid, and linolenic acid, respectively [66]. Both the wax and the oil significantly affected dough properties and of the organogel, such as firmness and thermal behavior. The highest organogel firmness was observed with sunflower wax and flaxseed oil. However, the main textural parameters (hardness, fracturability, spread factor) of cookie samples containing the wax–vegetable oil oleogel were not affected by different waxes and vegetable oils. Several cookies made with the oleogels showed similar characteristics to cookies made with a commercially available margarine. The network formed by the gluten present in the flour contributes more significantly to the cookie’s structural properties than the fat phase. Therefore, there is a high feasibility for the utilization of the organogel technology in real foods, such as cookies rich in unsaturated fats. The sensorial acceptance, as well as the texture and structural stability of cookies prepared with hazelnut oil gelled either with sunflower wax or beeswax instead of bakery shortening, were analyzed [68]. The textural properties and some physical attributes of the oleogel containing cookies resembled commercial bakery cookies. The consumer hedonic scores indicated even higher preference for the oleogel cookies, representing a high chance of acceptance from the consumers.

#### 2.3.2. Cakes and Baked Goods

The more aerated the structure of a cake, the better its quality, and the resulting porous structure is due to the incorporation of air during the whipping of the batter and because of baking the product. Low viscosity batters retain the air bubbles during whipping, leading to a low volume expansion of the final product. Cake batters are oil-in-water emulsions, the continuous aqueous phase containing dissolved or dispersed non-fat ingredients (flour, sugar, salt, yeast, and milk powder). For the aim of lowering the caloric value and the fat content of the cakes, watery oleogels were prepared—a reduction of saturated fatty acid contents and an increase in unsaturated fatty acids being obtained [72]. Oleic sunflower oil, cottonseed oil, and fat blend were gelled with 5% carnauba wax, water-free oleogels, as well as watery oleogels (emulsified) being prepared. Some physicochemical properties of oleogels, the rheological properties of the batters and the volume, structure, appearance, color, and sensory properties of cakes containing oleogel were also analyzed and compared to a reference. The sensorial analysis indicated that the panel considered the oleogel cakes acceptable, while the most preferred were the cakes containing oleogel of high oleic acid sunflower oil and cottonseed oil in equal amounts [72].

The solid fat reduction in aerated baked goods was also possible by introducing wax oleogels in the cake formulation. Canola oil gelled with carnauba wax influenced the cake batters’ viscosity and impaired them lower pseudo plastic properties. Different amounts of shortening were substituted by oleogels. The replacement levels influenced the textural parameters of the cakes, the oleogel containing cakes being firmer, but lower in volume. The tomographic analysis depicted well-structured cakes with a dense structure—the porosity and fragmentation index being negatively affected by the oleogel inclusion in the cake’s formulation. However, the air holding capacity of the batter was not affected at a replacement level of 50%. Therefore, it is possible to design cakes with lower saturated fat content than the shortening containing references by using wax containing oleogels. Overall, the use of oleogels mixed with shortening up to 25%, led to cakes with lower levels of saturated fatty acids, while the novel product did not display any loss in terms of quality [73].

Rice bran wax, beeswax, and candelilla wax were utilized to produce sunflower oil oleogels for shortening replacement in baked cakes. The result indicated that only beeswax oleogels are a good candidate for formulating cakes with properties comparable to the reference samples which contain shortening. Rice bran wax and candelilla wax oleogels formed more viscous cake batters than the beeswax oleogels, but less viscous than the control. The batters showed lower viscosity and less shear-thinning characteristics when shortening was replaced with studied oleogels. By introducing oleogels as ingredients of baked cakes, the levels of saturated fatty acids in the cakes were reduced from 58% to levels as low as 14%–17%. Beeswax oleogels utilization for shortening replacement led to cakes with lower hardness, comparable specific volume to the control sample, and a porous structure [74].

Sponge cakes were prepared having as ingredient β-carotene containing oil-in-glycerol (O/G) emulsion gels instead of bakery margarine. The oleogelator used for developing this emulgels was zein, a self-assembling amphiphilic protein, a by-product extracted from corn or maize. Prior to preparation, zein, and β-carotene were dissolved in heated glycerol. The mixture was homogenized (10,00 rpm for 3 min) and preheated soybean oil was also added at a specific volume fraction. The last step in the preparation of emulgels was cooling down to room temperature. The dispersed oil droplets are embedded in the matrix of glycerol phase, gelled using network of zein polymers, resulting oil in glycerol emulsion gels. The alternative margarine formulation displayed a ”spreadable” dominant behavior when the β-carotene concentration was increased. Sponge cakes were prepared from both commercial bakery margarine (rich in hydrogenated oils or saturated fats) and zein based β-carotene emulgels cake (low fat content, ~60%), with similar textural properties (firmness, cohesiveness, adhesiveness, and springiness) as the reference [69].

A high amount of fat is compulsory for gluten-free bakery products with satisfying physical characteristics. Gluten-free products often contain higher amounts of saturated fatty acids. Beeswax containing sunflower oil oleogels were proposed for reformulating the gluten-free aerated products. 55%, 70%, and 85% beeswax oleogel was mixed with shortening and batters and baked goods with properties comparable to those of the reference were obtained. As a result, it is possible to reduce the levels of saturated fats with 35% in gluten-free aerated products by using oleogel as ingredient, without affecting the textural properties [75].

Foam structured oleogels of sunflower oil with 4% hydroxypropyl methylcellulose were introduced in muffins composition to propose a healthier composition. The conventional shortening was replaced with oleogel on different levels (25%, 50%, 75%, and 100%). The resulting muffins batters displayed viscoelastic parameters different from the reference. The X-ray analysis revealed that muffin batters’ capability of entrapping air was also affected. The shortening replacement with hydroxypropyl methylcellulose oleogels was, however, possible at levels of up to 50%, without consequences over the textural parameters, volume, or porosity [77]. Canola oil gelled with ethyl cellulose, extruded at several ratios, was also assessed for utilization in laminated pastries [100]. A full replacement of margarine in muffins was also possible due to an oleogel formulated with high oleic sunflower oil and 4%, 7% or 10% monoglycerides. Besides lowering the saturated fats, the oleogel incorporation in the muffins reduced oil migration in the product by 50% [78].

#### 2.3.3. Bread

Recently, fats structured with ethyl cellulose polymers were analyzed in order to attain the functionality of shortenings used as ingredients in bakery. Different mixtures of palm stearin and soybean oil were proposed, the resulted composition presenting different final amounts of saturated fats. These were structured using cellulose, with different viscosity properties (EC7, EC20, EC50, and EC100) and 1% emulsifier (triglyceryl monostearate). The properties of the formed oleogels were assessed in search of the one which develops a stable and soft bread. The 100% replacing of the bakery shortening in bread with 4% EC100 oleogel with 30% degrees of saturation led to novel products with volume comparable to control, thus, a great amount of air bubbles incorporated in the dough. The oleogel structure led to firm bread with specific texture [76].

### 2.4. Meat Products

The numerous experimental results obtained by scientists who formulated meat products containing oleogels may represent a starting point for the meat industry to implement this alternative formulation for nutritional and technological considerations. Hardstock is generally considered a natural source of trans fats and processed meat products are also reported to contain on average 35% saturated fats, being a major responsible for the occurrence of cardiovascular disease among consumers [101]. Fats account for the structure and taste of meat products, its composition displaying binding properties which further influence the stability and texture of meat products. Since consumer preferences also depend on the quality parameters, it is highly desirable to maintain the properties of the classical meat product in the novel formulation.

#### 2.4.1. Frankfurters and Sausages

In an attempt to improve the nutritional properties and the fatty acid profile of meat products and frankfurters, oleogels were developed from canola, soybean or flaxseed oil with 10% ethyl cellulose (different molecular weight and viscosity, EC10, EC45, and EC100). The resulting gels maintained the chemical composition of the vegetable oil used, while transforming the oil into a structured material suitable for the replacement of saturated fats in food products. In fact, the strength of the resulting network was dependent on polymer molecular weight, the amount of ethyl cellulose used for structuring and the type of the vegetable oil. The 100% replacement of beef fat in frankfurters led to an oleogel containing product with similar textural properties as the reference [102].

Frankfurters reformulation was proposed using sunflower oil oleogels, structured with phytosterol mixture; a partial replacement was attained without affecting the organoleptic and physical properties of the products. Emulsions of the oleogels were also prepared for the aim of reducing the total fat amount of the final product. The phytosterols *γ*-oryzanol and *β*-sitosterol were used in 10% or 20% for the structuring of sunflower oil. Nine frankfurters samples were made with a 20% total fat content. Eight treatments were proposed for novel products, containing 10% pork back fat and 10% structured gels or emulsion of sunflower oil oleogels. The lipid oxidation and the pH of the oleogel containing frankfurters displayed comparable levels. The textural examinations showed no major quality differences between the oleogel containing frankfurters and the control. However, frankfurters containing oleogel emulsions registered lower chewiness, hardness, and gumminess. Sensory analysis showed the overall acceptability of every sample, not only the control, except for some samples containing emulsions of the oleogels [81]. The pork back fat reduction was also explored with oleogels structured with monoglycerides:phytosterols (3:1) and a 50% replacement of pork back fat with sunflower oil oleogel in frankfurter sausages was achieved. The interaction between monoglycerides and phytosterols displayed a synergism and a modified crystallization behavior; it led to strong network formation, higher values of hardness and gel strength; high values of storage modulus were obtained in comparison with the oleogel structured solely with monoglycerides. Also, the melting temperatures were lower compared to monoglycerides oleogels. The novel oleogel containing frankfurters and the references were similar in terms of cohesiveness and elasticity, but there were differences in terms of hardness, brittleness, gumminess, and chewiness, which had higher values for the control than the oleogel-substituted samples. The sensory evaluation revealed similar overall acceptance for the new frankfurter sausages, while no differences were measured in the oxidation levels [80].

A venison salchichon was prepared with olive oil oleogel emulsified with soy protein and water, instead of traditional pork meat, to result in a healthier product. Several salchichon samples were manufactured, the control being prepared from 75% lean venison and 25% pork meat. In the oleogel containing samples, the pork meat was replaced by oleogel in the following ratios—15%, 25%, 35%, 45%, and 55%; as expected, higher olive oil oleogel ratios determined an increase in monounsaturated fatty acid content. During ripening, the resulted samples were comparable in terms of physicochemical properties (pH, water activity, and losses), as well as instrumental color assessment; acidity index and lipid oxidation were in acceptable levels; overall, all studied salchichon samples were declared acceptable by consumers, while the samples with maximum of 25% of the pork meat replacement obtained the highest scores. It was concluded that the oleogel salchichon accepted by the consumers contains a double amount of oleic acid compared to the traditional salchichon composition [87].

#### 2.4.2. Meat Patties

Canola oil was gelled with the foam-structured template approach using hydroxypropyl methylcellulose, being evaluated as to whether it can totally or partially reduce the level of saturated fat in meat patties by replacing the beef tallow from the composition. The textural properties (firmness and work of shear) of the oleogels were analyzed, and higher values were obtained in comparison to the beef tallow. The values had a tendency to increase with increasing levels of hydroxypropyl methylcellulose percentage in the formulated oleogels. The cooking loss of the patties was significantly reduced for the samples that contained 50% and 100% hydroxypropyl methylcellulose oleogels. A soft texture was also reported. The highest overall acceptability of the samples on a sensory analysis was attained for the sample which contained 50% replacement of the beef tallow with oleogel. In conclusion, saturated fatty acids in the meat patties containing hydroxypropyl methylcellulose oleogels were significantly reduced to 15% compared to the beef tallow sample (42%), and the novel product presented good acceptability [83].

Healthier formulation was proposed for an extensively consumed meat product: the beef burger. The effect of two variables on the sesame oil oleogelation was analyzed, namely the organogelator concentration and the cooling temperature. The concentration of the beeswax used for oleogel formation was 5%, 7.5%, or 10%, and the cooling temperature used for the development of the oleogel structure was either room temperature (25 °C) or refrigeration conditions (4 °C). As reference samples, the extracted fats from beef flank and shank were considered. Acid and peroxide values, fatty acid composition, color, texture, thermal properties, and crystal morphology were studied. Then, 25% and 50% of animal fat in the beef burger was replaced by the oleogel, considered optimal after preliminary analysis (10% beeswax). Results indicated that acid value and thermal properties of the oleogels were affected by the beeswax concentration, and lipid oxidation increased significantly. A 50% reduction in the quality of the textural parameters of raw burgers was registered. Thus, hardness, gumminess, and chewiness of the raw burgers were affected by the replacement of animal fats with the healthier alternative represented by the oleogel. There were some positive results, including the color while cooking the burger, a 1.6% decrease of fat absorption, and a 11% reduction of cooking loss [84].

Linseed oil was gelled using an equimolar mixture of *γ*-oryzanol and *β*-sitosterol. The oleogels were added in the formulation of pork patties in two different amounts and they were analyzed in comparison to a commercial hamburger formulation. Sous vide cooking procedure was applied to hamburgers and a compression test was performed to evaluate the hardness and chewiness. The hamburger formulated with 25% oleogel and subcutaneous pork fat displayed similar textural parameters; it was also secondly preferred in the sensory evaluation, after the reference hamburger. The cost evaluation of producing oleogel based hamburgers were also analyzed, resulting that for a 25% containing oleogel there are no extra costs for purchasing the ingredients [90].

In order to enrich the composition of pork burgers with polyunsaturated fatty acids, a mixture of vegetable oils with various fatty acid composition was designed, as following: 44.39% olive oil, 37.87% linseed oil, and 17.74% fish oils. The mixture was gelled with either 11% ethyl cellulose or 11% beeswax, with or without the addition of 2% curcumin. Ethyl cellulose oleogel was prepared by the dispersion of the mixture, followed by sonication and cooling down to room temperature. Beeswax oleogel was prepared by heating the oil and 11% beeswax under constant stirring, sonication and storage at 3 °C after staying for 30 min at room temperature, in darkness. Five different samples of burgers were prepared by mixing the minced frozen meat and fatty ingredients. Further analyses on oleogel containing food products are of utmost importance for upscaling the novel processes. The effect of refrigeration temperature and the cooking of the oleogel containing pork burgers were analyzed in terms of sensory, technological properties, oxidation, and microbiological stability. The novel low-fat burgers (7.5%) displayed a softer texture than the reference, a higher antioxidant ability in the burgers containing curcumin. However, thermal treatment promoted lipid oxidation for all of the analyzed samples, regardless of the composition or curcumin presence. The sensory analysis revealed a preference for the classic burger, but the beeswax oleogel containing formulation also presented encouraging acceptability values [92].

#### 2.4.3. Pâtés

The pork liver pâtés composition was reformulated with the use of oleogels, in an attempt to improve the nutritional profiles and to reduce the content of trans and saturated fats that may occur from the meat implied in the preparation. Ethyl cellulose and sorbitan monostearate or beeswax oleogels were prepared using a lipid composition resulting from mixing olive, linseed, and fish oil, designed for a unique and desired fatty acid composition. Due to the oleogelation, the mixture of oils displayed both solid and lipid functionality and could be used as animal fat replacer in food products. Ethyl cellulose oleogel was softer and more deformable, displaying flexibility in the conformation, and high thermal stability. Neither the stability of the emulsion, nor the appearance or texture were changed by the inclusion of oleogel in the formulation; the oleogel emulsion had the capacity to replace pork back fat. As a negative aspect, the oxidation processes were more prominent and occurred even during refrigeration storage for the sample with the highest amount of pork back fat substituted by ethyl cellulose oleogel. The use of 11% beeswax oleogel did not affect the sensory parameters assessed, while the ethyl cellulose oleogel containing products were negatively appreciated in a directly proportional manner with the substitution levels. As a conclusion, the beeswax oleogels are better candidates for the reformulation of pork liver pâtés due to improvement of their nutritional profile; in this study, the ethyl cellulose oleogel was less suitable for the intended purpose. The beeswax containing products are comparable with the classic pork liver pâtés in terms of consumer’s acceptance [91].

### 2.5. Dairy

Although milk contains saturated fatty acids and trans fatty acids, it also contains valuable components beneficial for consumer’s health, like calcium, conjugated linoleic acid, sphingomyelin, butyric acid, ether lipids, β-carotene, and vitamins A and D. For these reasons, formulating alternative products will be quite challenging in terms of consumers’ acceptance. Fats in dairy products play roles in flavoring the final products in a unique and specific manner, but they are also important for texture and structure formation. Milk fat is composed of numerous triacylglycerol and their ability to crystalize make them indispensable for some applications [103].

In ice-cream, the type of fat chosen for the mix preparation, imparts some properties to the end product. The sensory properties, such as flavor, creaminess, and taste are dependent on the fats melting down during serving. The emulsifier from the ice-cream destabilize the fat in order to aggregate and capture air bubbles incorporated in the composition during freezing. The fat crystal size and shape also influence the air bubble stabilization.

Cream cheeses are acid coagulated cheeses which can be produced from milk fat or vegetable fat, the second option being preferred for economical, nutritional, and technological reasons, but it is important that fatty acids present similar composition and functionality to milk fat. Fat and particle size distribution of fat are responsible for texture properties like consistency, appearance, and shape formation during ripening.

#### 2.5.1. Cream Cheese

The simultaneous manufacturing of a cream cheese by oleogelation of the containing fat phase was proposed in order to reduce the saturated fat amount and, simultaneously, increase the unsaturated fats content of the product. High oleic soybean oil and regular soybean oil were gelled with 10% rice bran wax or ethyl cellulose in the presence of other non-fat ingredients. The new formulations were compared to both full-fat and fat-free commercial cream cheese products and the textural analysis revealed similarities in terms of hardness, spreadability, and stickiness values. However, the ethyl cellulose oleogel cheese cream samples exhibited reduced values for adhesiveness, compared to the full-fat control. Confocal laser scanning microscopy analysis demonstrated microstructural suitability for the incorporation of oleogels into a cream cheese product, but also similarities in the fat globule size. Further research was recommended in order to develop an oleogel cream cheese spread that displays similar storage modulus throughout the entire temperature range and also suitable for other applications, such as heating, cooking, and baking [104].

Knowing the reported success of rice bran wax oleogels incorporation in the cheese cream products, other formulations have been proposed. The effects of processing on the oil’s oxidative stability and the tocopherol content were also investigated so as to obtain a better insight of the nutritional quality of the product. High-oleic soybean oil was gelled with rice bran wax, concomitantly with the preparation of the cream cheese product. An un-gelled cream cheese sample and two commercial cream cheese products were used as controls. High-performance liquid chromatography analysis displayed comparable values for α-tocopherol of the oil and the oleogel samples, the latter showing a lower total tocopherol content. No significant differences were observed between the total tocopherol contents of the oleogel cheese cream sample and the un-chilled cream cheese product; the amount of all three-tocopherol isomers remained constant during 14 days of storage. The assessment of the oxidative parameters showed higher amounts of volatile compounds in the oleogel samples compared to the oil and also a minor difference in the content of the volatile compound between the oleogel sample and the reference. The results show minimal degradation of vegetable oleogels due to the thermal processing and storage. This result offers information necessary for the practical application of oleogels in dairy products [93].

Milk fat contained by the cheese products was replaced by soybean oil gelled using rice bran wax or sunflower wax, and the final concentrations of waxes in the cheese products was as low as 0.5% or 1%. The rheological and thermo-mechanical analysis resulted in comparable properties, such as hardness, storage modulus, and meltability, between the products based on wax oleogel and the control samples obtained with commercial milk fat [94]. The utilization of gelled oil in processed cheese products resulted in a less expensive product, with a reduced content of milk fat, no added chemicals or processing agents and also improved physicochemical properties of low solid fat cheese products. There are significant differences in how the fatty acids of vegetable oils interact with the protein network in comparison to milk fat’s normal interactions. Further research into this phenomenon will support a better understanding of milk protein–oleogel matrix formation and the effects on replacing milk fats with oleogels in a food system and the consequences over properties such as oil-binding capacity, texture, and melting characteristics.

#### 2.5.2. Ice-Cream

Different oleogels were used for formulating ice-cream with lower saturated fat content [95,96,97]. Rice bran wax oleogel was used for 15% fat containing ice-cream formulation, this research also revealing that glycerol monooleate was the most suitable emulgator for the fat network formation in this system using the continuous freezing technique. The ice-creams produced with oleogel containing 12% mixture of sterols successfully replaced milk cream in 4% or 8% fat containing ice-cream. The characteristics of the novel ice -cream were comparable to those of the samples containing milk cream, and it presented even better overrun and melting parameters. In conclusion, the application of organogels in ice-creams as milk cream substitutes might be a successful approach in order to obtain healthier products [97].

## 3. Future Trends of Oleogel Application in Food

Controlled or delayed release of nutraceuticals and pharmaceuticals added in foods in proper concentrations can be achieved by means of oleogelation. Some fat-soluble molecules such as β-carotene, lycopene, co-enzyme Q10, docosahexaenoic acid, eicosapentaenoic acid, conjugated linoleic acid, tannins, plant sterols, and isoflavone are consumed in very low amounts. Their consumption provides medical or health benefits, including some diseases prevention and treatment; that is why encapsulation or delivering by means of oleogels is desired, followed by their incorporation in functional foods.

The delivery of lipid-soluble molecules from the oleogel is influenced by some properties of the oleogel, like its texture and structure, which depend on the type and amount of oleogelators implied in the process [48]. When delivery systems are designed, it is important to bear in mind that the structure of the delivering material has a strong influence on digestibility and nutrient bioaccessibility. The majority of the lipid digestion and absorption takes place in the stomach and slowing intestinal lipolysis seems to help combat obesity [105]. The bioavailability of the compounds depends on the micellarization and solubilization of the hydrophobic bioactive molecules. Micelles occur during digestion when the vegetable oil migrate out of the oleogel and these micelles spread throughout the physiological environment, serving as vehicles for the bioactive molecules [106].

Moreover, oleogels seem to increase the bioavailability of lipid soluble molecules. The micellarization rates of β-carotene from canola oil or from a 12-HSA canola oil gel during in-vivo digestion were determined and the results showed that the maximum release of β-carotene from oil occurred between 0 and 30 min of intestinal digestion, while for oleogel, the maximum release of β-carotene was in between 30 and 75 min [71]. Zein-based emulsion oleogels containing β-carotene were also designed. Both glycerol and zein were good vehicles for the incorporation and color-stabilization of β-carotene molecule. The zein oleogel assured the protection and retention of the bioactive β-carotene and hindered the lipid oxidation [69].

Curcumin is a water insoluble nutraceutical compound which demonstrated many health promoting effects; thus, curcumin-controlled delivery was proposed by forming oleogels of medium chain triacylglycerols, canola, coconut or corn oils with monostearin as a GRAS organogelators, with the addition of Span 20. The oleogel contained 2.6% curcuminoids and the bioaccessibility was higher for the oleogel than for curcuminoids powder dispersed in water. The bioaccessibility was also 5 times higher for the fasted state than for the fed state. The oleogel formation was also highly efficient in preventing the precipitation of curcuminoids during storage [107].

Oleogels were used for the deep-frying processing of instant noodles. Soybean oil gelled with carnauba wax was used in dried and precooked fried noodles instead of a medium rich in saturated fat, such as palm or soybean oil. The noodles fried in oleogels absorbed less oil, and there were no negative effects related to texture. The content of saturated fatty acids of the noodles precooked in oleogel was 19% lower compared to the palm oil-fried product. The use of oleogels presents possible opportunities to formulate healthier fried goods by improving the composition of the final product. However, in order to become a real practice, studies should be conducted on different oleogel mediums and different frying processes [98].

## 4. Conclusions

In conclusion, recent policy changes that impose a removal of trans fats from food products and limitations in the consumption of saturated fats, along with the rising concerns among consumers about the negative effect of fats and the ecological damages caused by the intense palm oil usage, led to numerous research in the field of fat reformulation. Innovations in reformulating the fat-containing food products are based on the introduction of oleogels in the food matrix or on the formation of some blends containing the original fat source and the novel oleogels.

Despite the promising results and formulations, the wide range of oleogelation techniques and organogelators, the similarities between the physical properties of oleogels and those of fat-containing food products, in spite of the nutritional value brought by the usage of vegetable oils and despite the positive results of sensorial analysis and hedonic tests of oleogel containing food products, there are still no commercial available oleogel containing food products.

Therefore, there is a great need to further exploit the impact of different processing parameters specific to some food products over the oleogel properties, also to promote the benefits of oleogels usage among food producers, as well as the nutritional benefits and the impact on the environment in order to increase the consumers’ acceptance and consumption of oleogel containing food products.

## Figures and Tables

**Table 1:** Topics of books and selected reviews published on oleogelation subject.

Topic	References ^1^
Systematically presenting (up to the publishing date) the available oleogels and their use as conventional fat replacers	**Books**
	Marangoni and Garti 2018 [30]; Patel 2018 [31]; Patel 2015 [32] Marangoni and Garti 2011 [33]
	**Review type articles**
	Martins et al. 2018 [22]; Pehlivanoğlu et al. 2018 [3]; Chaves et al. 2018 [34]; Singh et al. 2017 [35]; Patel and Dewettinck 2016 [19]; Co and Marangoni 2012 [20]; Dassanayake et al. 2011 [36]; Rogers 2009 [37]; Hughes et al. 2009 [18]; Pernetti et al. 2007 [17]
Proteins as agents for oil structuring	Scholten 2019 [38]
Polysaccharides as agents for oil structuring	Davidovich-Pinhas 2019 [39]
Oleogels used in bakery products	Demirkesen and Mert 2019 [40]
Delivery of bioactive compounds by designing gel structures in water and oil phases	Mao et al. 2019 [41]
Replacement of conventional fat in baked products	Colla et al. 2018 [42]
Hydrocolloids as agents for oil structuring	Patel 2018 [43]
Examining Hansen solubility parameters to provide insight into what types of molecules might be able to structure vegetable oils—bottom-up research in the quest for new oleogels	Rogers 2018 [44]
Edible polymer oleogels characterization	Davidovich-Pinhas et al. 2016 [45]
Oil structuring techniques applied in order to increase the fat quality of meat products	Jimenez-Colmenero et al. 2015 [46]
Oil structuring techniques for oral delivery of lipid soluble molecules	Esposito et al. 2018 [47]; O’Sullivan et al. 2016 [48];

^1^ Reverse chronological order inside each topic.

**Table 2:** Overview of oleogels used in food: short description, structuring strategy, and a brief conclusion.

Analyzed Oleogels	Structuring Strategy	Brief Conclusion	Reference
**Breakfast Spreads**			
Soybean oil, almond oil, canola oil, corn oil, flaxseed oil, grapeseed oil, peanut oil, pumpkin seed oil, safflower oil, sesame oil, sunflower oil, walnut oil, individually gelled with 3%, 5%, or 7% sunflower wax	Direct dispersing, followed by cooling for 60 min in ice bath, then storage in refrigeration conditions (4–5 °C)	100% fat replacement of commercial margarines	Hwang et al. [49]
Soybean oil gelled with 2–6% sunflower wax, rice bran wax, candelilla wax	The oleogel was mixed with lecithin, and monoglycerides; the water phase (salt, citric acid, calcium disodium, potassium sorbate, skim milk) was added in the prepared oleogel phase by stirring at 3000 rpm, 7 min	100% replacement of hydrogenated soybean oil with a 2–6% sunflower wax soybean oil oleogel	Hwang et al. [50]
Sunflower oil gelled with 2% shellac	The direct dispersing method followed by cooling below its crystallization temperature	100% replacement of hydrogenated oils with 2% shellac oleogel forming an emulsion up to 60% water-in-oil	Patel et al. [51]
Virgin olive oil gelled with 3.75%, 4.5% beeswax, and Tween 20 or Tween 80, forming a water-in-oil emulsion	Simultaneous formation of oleogel and emulsification, followed by cooling down to room temperature overnight	100% replacement of hydrogenated oils with a water-in-oil emulsion of 3.75% or 4.5% beeswax virgin olive oil oleogel	Öğütcü et al. [53]
Virgin olive oil gelled with 3%, 7%, 10% carnauba wax or 3%, 7%, or 10% monoglycerides	The direct dispersing method followed by cooling down at ambient temperature	Margarine, with 7% monoglycerides containing virgin oil oleogel was similar to a commercial breakfast spread	Öğütcü and Yılmaz [52]
High oleic sunflower oil was gelled with 6%, 10%, or 14% Myverol monoglycerides)	Dispersing of organogelator in the oil, followed by heating and constant stirring for 30 min and cooling down at room temperature	The hardness value for the oleogels was similar to margarine, the rheological behavior indicated similarity; whereas the adhesiveness and cohesiveness values were different	Palla et al. [60]
**Confectionery**			
Rapeseed oil gelled with 2% shellac wax	The direct dispersing method, oleogel used as an ingredient to chocolate paste formation	Oleogel displayed oil binder action; 27% of palm oil was replaced with oleogel in chocolate pastes	Patel et al. [51]
Pomegranate seed oil gelled with 5% saturated monoglycerides, beeswax, and propolis wax	The direct dispersing method followed by cooling down at room temperature	50% replacement of palm oil with oleogel in chocolate spreads	Fayaz et al. [58]
Corn oil structured by 10% monoglyceric stearate, 10% *β*-sitosterol/lecithin (8:2), or 10% ethyl cellulose	The direct dispersing method followed by stirring (400 rpm) and heating until completely dissolved, storage at 4 °C	Monoglyceric stearate corn oil oleogel replacing 100% cocoa butter in dark chocolate	Li and Liu [61]
**Analyzed Oleogels**	**Structuring Strategy**	**Brief Conclusion**	**Reference**
**Confectionery**			
Sunflower oil gelled with 10% or 25% mixture of 1:1 *γ*-oryzanol and *β*-sitosterol	The direct dispersing method followed by storage at ambient temperature	2.5% or 14% of sunflower oil oleogels were included in praline system in different layers; the oil migration was 50% reduced	Wendt et al. [62]
Soybean oil gelled with 1%, 3%, or 6% monoglycerides, and also with 6%, 8%, or 10% of a mixture (1:1) of sorbitan tri-stearate and lecithin	The direct dispersing method under stirring conditions, followed by crystallization at ambient temperature for monoglycerides oleogels and 5 °C under static conditions for 24 h, for the sorbitan tri-stearate and lecithin oleogels	3% and 6% monoglycerides soybean oleogels and 8% and 10% sorbitan tri-stearate and lecithin oleogels displayed similar properties to those of confectionery filling fats in praline model system	Si et al. [63]
Rice bran oil gelled with 1.5%, 2%, 2.5%, 3%, or 3.5% beeswax for 17%, 33% and 50% palm oil replacement in hazelnut fillings	The direct dispersing method followed by subsequently cooling to 5 °C, at 1.0 °C/min cooling rate	Efficient replacement of 17% of palm oil with oleogel in hazelnut filling; oil binding properties noticed	Doan et al. [64]
**Pastry**			
Canola oil gelled with 3% and 6% candelilla wax	The direct dispersing method followed by cooling down to room temperature	30% replacement of shortening in cookies with 3% and 6% candelilla wax oleogel and 60% replacement with 6% candelilla wax oleogel	Mert and Demirkesen [65]
Olive oil, flaxseed oil and soybean oil, gelled individually with 2–10% of different waxes: sunflower wax, rice brain wax, beeswax, and candelilla wax	The direct dispersing method and cooling down at ambient temperature	Cookies containing oleogels instead of margarines can be formulated without altering the dough and the cookies properties	Hwang et al. [66]
Canola oil gelled with 3% or 6% candelilla wax	The direct dispersing method under agitation for 10 min, followed by cooling down to room temperature overnight	Shortening replacement in cookies. The saturated fatty acids of cookies were reduced with 8–10% due to oleogel use	Jang et al. [67]
Hazelnut oil gelled with 5% sunflower wax or 5% beeswax	The direct dispersing method followed by cooling down to ambient temperature overnight	Commercial bakery shortening was 100% replaced	Yilmaz and Öğütcü [68]
Soybean oil gelled by 3% zein forming an emulsion in glycerol with β-carotene fortification (0–0.045%)	Zein and β-carotene were dissolved in heated glycerol and the mixture was homogenized (10,000 rpm for 3 min), preheated soybean oil being also added at a specific volume fraction, followed by cooling down to room temperature	Bakery margarine was 100% replaced in sponge cake by zein glycerol oleogel emulsion fortified with β-carotene, resulting similar textural properties between the novel cake and the reference	Chen et al. [69]
Canola oil was gelled with 1% hydroxypropyl methylcellulose or 1% methylcellulose	Foam templating approach and freeze drying	50% and 75% replacement level of shortening in sandwich cookie cream were similar to creams containing 40% fat content	Tanti et al. [70]
**Analyzed Oleogels**	**Structuring Strategy**	**Brief Conclusion**	**Reference**
**Pastry**			
The ethyl cellulose oleogels were not formed prior to cookie formation	The ethyl cellulose was added as ingredient dispersed in the oil during baking and, upon cooling, gelled the fat phase, which could slow oil leakage from the cookie	Low-fat, low-saturated cookies were prepared with fat alternative—Coasun shortening and 3% or 5% ethyl cellulose, which hindered oil leakage in the product	Stortz et al. [71]
Oleogels with or without 18% water were produced from high oleic sunflower oil, cotton seed oil and 5% carnauba wax	5% carnauba wax was added to different fat blends to form oleogels or emulsified oleogels, followed by the addition of specific cake ingredients	Cake formed with oleogel containing 50% cotton seed oil and 50% high oleic sunflower was the most acceptable, being rich in unsaturated fatty acid and low in saturated fatty acid	Pehlivanoglu et al. [72]
Canola oil was gelled with 10% carnauba wax	Direct dispersing and continuously agitation with a laboratory stirrer, cooling down at room temperature	25–50% of shortening replacement in cakes	Kim et al. [73]
Sunflower oil gelled with 10% rice bran wax, or 10% beeswax, or 10% candelilla wax	Direct dispersing method with agitation, followed by cooling down at room temperature	Beeswax oleogel can replace 100% shortening in cakes; cakes low in saturated fat and high in unsaturated fat without losses of quality parameters were obtained	Oh et al. [74]
Sunflower seed oil gelled with 10% beeswax blended with 20%, 40%, 70% or 85% shortening	Direct dispersing method	45%, 30%, and 15% replacement of shortening in gluten-free cakes	Demirkesen and Mert [75]
Palm stearin and soybean oil mixture gelled with 1%, 2%, 3%, 4%, or 6% ethyl cellulose (EC7, EC20, EC50, EC100) and addition of 1% emulsifier	The fat phase was heated, and ethyl cellulose and emulsifier were dispersed, then rapidly cooled in an isopropanol-water ice bath (–40 °C) under constant stirring (200 r/min for 2.5 min), until gel formation	100% replacement of bakery shortening with 4% EC100 oleogel from palm stearin (30% degrees of saturation) + soybean oil mixture in stable soft textured bread	Ye et al. [76]
Sunflower oil gelled with 4% hydroxypropyl methylcellulose blended with 25%, 50%, 75%, and 100% shortening	Foam-template approach	50% replacement of shortening in muffins	Oh and Lee [77]
High oleic sunflower oil gelled with 4%, 7%, or 10% monoglycerides	Direct dispersion of monoglycerides in the heated oil under magnetic agitation, followed by cool down to room temperature	Replacement of commercial margarine in muffins by optimized oleogels and 50% reduction of oil migration as compared to control	Giacomozzi et al. [78]
Sunflower wax, shellac wax, and beeswax were added to the halva composition at different levels (1%, 3%, or 5%)	Tahini and sugar syrup mix was prepared at isothermal conditions and the waxes were dispersed in the mixture	100% replacement of hydrogenated palm stearin which is used as additive in the production of halva	Öğütcü et al. [79]
**Analyzed Oleogels**	**Structuring Strategy**	**Brief Conclusion**	**Reference**
**Pastry**			
Camellia-oil based oleogels were structured with tea polyphenol-palmitate particles and 1.5%, 2.5%, 3.5%, or 4.5% citrus pectin	The indirect method of oleogel formation starting from the formation of an emulsion	Butter was replaced in cakes with tea polyphenol-palmitate and citrus pectin camellia-oil oleogels; the 1.5% and 2.5% citrus pectin oleogels were sensory acceptable	Luo et al. [28]
**Meat Products**			
Sunflower oil gelled with 20% mixture of monoglycerides and phytosterols (ratios of 1:1, and 3:1)	Direct dispersion of the oleogelators in the oil, followed by cooling down at ambient temperature	50% replacement of pork back fat in frankfurter with sunflower oil gelled with monoglycerides: phytosterols (3:1)	Kouzounis et al. [80]
Sunflower oil gelled with 10%, or 20% *γ*-oryzanol and *β*-sitosterol	*γ*-oryzanol and *β*-sitosterol were added in oil at ambient temperature. The solution temperature was raised and maintained at 90–120 °C for 30 min, under constant stirring conditions, followed by cooling	Oleogel successfully replaced 50% of fats from pork back fat in frankfurter formulation	Panagiotopoulou et al. [81]
Linseed oleogel gelled with 8% beeswax	Direct dispersion of the beeswax in the oil, followed by cooling down to room temperature	25% and 50% of pork back fat replaced by oleogel in frankfurters without affecting the texture	Franco et al. [82]
Canola oil gelled with hydroxypropyl methylcellulose	Foam structuring approach	50% replacement of beef tallow in meat patties had overall acceptability	Oh et al. [83]
Sesame oil gelled with 5%, 7.5%, or 10% beeswax	Direct dispersion of the beeswax in the oil followed by cooling down at 4 °C	Up to 50% replacement of fats beef flank and shank with 10% beeswax oleogel in burgers	Moghtadaei et al. [84]
High oleic sunflower oil gelled by pork skin	Pork skin (cooked 40 min at 80 °C, and then comminuted in a blender), water and high oleic sunflower oil were mixed in the ratio of 1.5: 1.5: 1	Replacement of 50% pork back fat in bologna sausages	da Silva et al. [85]
Linseed oil was gelled by a mixture of *γ*-oryzanol and *β*-sitosterol or beeswax (8%)	Direct dispersion of the oleogelators in the heated oil, followed by cooling down	Fermented sausages were prepared with two oleogels at two levels of replacement (20% and 40%) of pork back fat, quality changes (sensory, pH, color) being noticed; however, encouraging results were obtained, fatty acid profile being improved	Franco et al. [86]
Olive oil gelled with soy protein concentrate and mineral water andreplacing 15%, 25%, 35%, 45%, and 55% of the pork meat	Olive oil, soy protein concentrate, and mineral water in a 10:1:8 mixture were emulsified	25% of the pork meat replaced with oleogel in salchichon sausages resulted in a novel product, comparable with the reference	Utrilla et al. [87]
**Analyzed Oleogels**	**Structuring Strategy**	**Brief Conclusion**	**Reference**
**Meat Products**			
Canola oil was gelled with 8%, 10%, 12%, and 14% ethyl cellulose with no other addition or with 1.5% and 3% sorbitan monostearate	The organogelators and the oil were heated and reached the target temperature of 140 °C, ~50 min, followed by a 10-min holding period in the oven	Using an organogel prepared with 8% ethyl cellulose and 1.5% or 3.0% sorbitan monostearate resulted in a hardness value similar to that of beef fat by both sensory and texture profile analysis of frankfurters	Barbut et al. [88]
Beef fat, rendered beef fat, canola, soy and flaxseed oils were gelled with 10% ethyl cellulose with viscosity of 10 cP and 5% sorbitan monostearate	The organogelators and the oil were heated and reached the target temperature of 140 °C, ~50 min, followed by a 10-min holding period in the oven	When beef fat was introduced as lipid phase of the organogel, the hardness of meat batters was higher; a significant difference between the fast cooking rate products prepared with regular vs. organogel beef fat. Meat batters containing organogels prepared from canola oil showed the opposite effect, being softer	Barbut et al. [89]
Linseed oil gelled with 8% mixture of *γ*-oryzanol and *β*-sitosterol	Direct dispersion of the oleogelators in the heated oil, followed by cooling down	25% and 75% replacement of subcutaneous pork fat in meat patties.No differences between the patties produced with oleogel and the control, in terms of textural parameters (hardness, cohesiveness, and chewiness)	Martins et al. [90]
A mixture of olive oil, linseed oil, and fish oils gelled with 11% ethyl cellulose and 3.67% sorbitan monostearate or 11% beeswax	Ethyl cellulose oleogel was prepared by dispersion of the mixture, sonication, and cooling. Beeswax oleogel was prepared by heating the oil and 11% beeswax under constant stirring	15% pork back fat reduction in pâtés with beeswax oleogel; sensory test revealed that there were no significant changes of any of the parameters evaluated	Gómez-Estaca et al. [91]
A mixture of olive oil, linseed oil, and fish oils gelled with 11% ethyl cellulose and 3.67% sorbitan monostearate or 11% beeswax	Beeswax oleogel was prepared by heating the oil and 11% beeswax. Ethyl cellulose oleogel was also prepared by dispersion. Both strategies included the incorporation of curcumin (0.2%) during oleogel preparation, sonication, and cooling	Pork burgers formulated with beeswax oleogel presented adequate technological properties and good overall sensory acceptability. Curcumin effectively reduced the lipid oxidation process derived from chilled storage or cooking but conducted to reduced sensory acceptance	Gómez-Estaca et al. [92]
**Dairy**			
High oleic soybean oils gelled with 10% rice bran wax	The oleogel was formed in the development of the cream cheese product	The results showed minimal degradation of vegetable oleogel cream cheese due to the thermal treatment and storage	Park et al. [93]
**Analyzed Oleogels**	**Structuring Strategy**	**Brief Conclusion**	**Reference**
**Dairy**			
Rice bran wax or sunflower wax gelled soybean oil in 0.5% or 1% concentration in cheese	Direct dispersion of waxes in oil under stirring conditions followed by immediately transfer to a refrigerator at 4 °C	The oleogel utilization resulted in 20–22% reduction of the saturated fat content compared to the commercial cheese product formulation	Huang et al. [94]
High oleic sunflower oil was gelled with 10% rice bran wax, candelilla wax, or carnauba wax	Direct dispersion of waxes in the heated oil followed by cooling down	Rice bran wax was preferred as oleogelator in ice-cream application; when used, greater rates of meltdown and less fat destabilization were obtained	Zulim Botega et al. [95]
High oleic sunflower oil gelled with 10% rice bran wax	The oleogel ingredients were added to the mixture of ice-cream, which was pasteurized, homogenized, cooled, aged, frozen, and hardened	Oleogel was suitable for ice-cream formulation, but the structure formed by the oleogel system resulted in the product collapse	Zulim Botega et al. [96]
Sunflower oil was gelled with 8% or 12% mixture of phytosterols and *γ*-oryzanol	The oleogel ingredients were added to milk during heating, before adding the solid ingredients. The total fat content was either 4% or 8%	Ice-creams produced with oleogel containing 12% gelators showed similar or even better quality compared to the milk cream containing reference	Moriano et al. [97]
**Additional Applications**			
Soybean oil gelled with 5% or 10% carnauba wax	Oleogels used as alternative to deep-fat frying medium containing high saturated fat, for dried and precooked noodles	The samples fried in the oleogels absorbed ~16% less oil, with any negative effects on the noodle texture. Saturated fatty acids content of the oleogel-fried noodles were significantly lower (19%), compared to the palm oil-fried noodles (54%)	Lim et al. [98]
Canola oil gelled with 2% of 12-hydroxi stearic acid	12-hydroxi stearic acid was dispersed into oil and the mixture was heated. Gel was formed upon cooling	Release of β-carotene during digestion, revealing potential use for delivery of nutraceutical or biological active compounds and their controlled release	Stortz et al. [71]
